# Exposure to Hexachlorobenzene during Pregnancy and Children’s Social Behavior at 4 Years of Age

**DOI:** 10.1289/ehp.9314

**Published:** 2006-11-06

**Authors:** Núria Ribas-Fitó, Maties Torrent, Daniel Carrizo, Jordi Júlvez, Joan O. Grimalt, Jordi Sunyer

**Affiliations:** 1 Center for Research in Environmental Epidemiology, Institut Municipal Investigació Mèdica (IMIM), Barcelona, Spain; 2 Àrea de Salut de Menorca, IB-SALUT, Menorca, Spain; 3 Department of Environmental Chemistry; IIQAB-CSIC, Barcelona, Spain; 4 Universitat Pompeu Fabra, Barcelona, Spain

**Keywords:** behavior, children, cord serum, HCB, hexachlorobenzene, hyperactivity, prenatal exposure, social competence

## Abstract

**Background:**

Hexachlorobenzene (HCB) is an organochlorine chemical that has been used in agriculture and industrial processes. Behavioral impairment after HCB exposure has been described in animal models, but little information is available in humans.

**Objectives:**

Our goal was to study the association of prenatal exposure to HCB with the social behavior of preschool children.

**Methods:**

Two birth cohorts in Ribera d’Ebre and Menorca (Spain) were set up between 1997 and 1999 (*n* = 475). The California Preschool Social Competence Scale and the Attention-Deficit Hyperactivity Disorder (ADHD) were scored by each 4-year-old child’s teacher. Organochlorine compounds were measured in cord serum. Children’s diet and parental sociodemographic information were obtained through questionnaire.

**Results:**

Children with concentrations of HCB > 1.5 ng/mL at birth had a statistically significant increased risk of having poor Social Competence [relative risk (RR) = 4.04; 95% confidence interval (CI), 1.76–9.58] and ADHD (RR = 2.71; 95% CI, 1.05–6.96) scores. No association was found between HCB and the cognitive and psychomotor performance of these children.

**Conclusions:**

Prenatal exposure to current concentrations of HCB in Spain is associated with a decrease in the behavioral competence at preschool ages. These results should be considered when evaluating the potential neurotoxicologic effects of HCB.

Production and intensive use of organochlorine chemicals such as hexachlorobenzene (HCB) in agriculture and industrial processes in the past have led to widespread contamination of the environment. At present, HCB is still formed as a by-product during the manufacture of some chlorinated solvents (U.S. Environmental Protection Agency 1975). The main intake of organochlorine compounds (OCs) in humans is through diet and, in unusual circumstances, by inhalation (Sala et al. 1999b). Because of its lipophilic and chemically stable character, HCB can be found throughout the environment (Barber et al. 2005). Newborns are exposed across the placenta and by breast-feeding (Huisman et al. 1995; Rhainds et al. 1999).

HCB has a broad range of toxic effects on experimental animals, including immunotoxicity (Michielsen et al. 1999), disturbance of the thyroid function, and neurotoxicity (Agency for Toxic Substances and Disease Registry 2002). In rodents, HCB was detected in the brains of fetuses and pups after maternal exposure to the chemical (Courtney and Andrews 1985). Neurologic effects of HCB have been reported in victims of the poisoning incident in Turkey (Peters et al. 1982) and in short-term as well as in chronic exposure studies in various animal species (Courtney 1979; De Matteis et al. 1961). HCB has also been described as a potential behavioral teratogen. Rodent litters maternally exposed to HCB showed an increased risk of presenting behavioral alterations such as hyperexcitability and hyper-activity (Bleavins et al. 1984; Goldey and Taylor 1992; Lilienthal et al. 1996).

Unusually high atmospheric concentrations of HCB were found in the population of a rural village of 5,000 inhabitants in the vicinity of an electrochemical factory (Flix, Ribera d’Ebre, Spain) (Sala et al. 1999a). The factory, built in 1898, has been producing chlorinated solvents for four decades. Adult inhabitants studied in 1994 had the highest serum HCB levels ever found (mean = 36.7 ng/mL) (To-Figueras et al. 1997), and levels of HCB in the cord serum of newborns from this population studied in 1999 were among the highest ever reported (Sala et al. 2001). High concentrations of HCB were also found in newborns from Menorca (Carrizo et al. 2006). Menorca is one of the Balearic Islands in the northwest Mediterranean Sea, which has no local pollution sources.

Our aim in this study was to follow up the children from the Ribera d’Ebre and Menorca cohorts to assess the association of prenatal and postnatal exposure to HCB and social behavior of children at 4 years of age.

## Population and Methods

### Study participants

For the Ribera d’Ebre cohort we recruited all singleton children born in the main hospital of the study area between March 1997 and December 1999 (Ribas-Fito et al. 2003). The study area included the village of Flix and all other towns from the same administrative health area. One hundred two children were enrolled and 70 provided complete outcome data for children 4 years of age (68.6%) and OCs measured at birth. The Menorca cohort was set up in 1997 within the Asthma Multicenter Infants Cohort study (Polk et al. 2004 ) and recruited all women presenting for antenatal care over 12 months starting in mid-1997. Four hundred eighty-two children (94% of those eligible) were subsequently enrolled and 422 (87.5%) provided complete outcome data up to 4 years of age. Among these, 405 (84%) had OCs measured in cord serum. This study was approved by the ethics committee of the Institut Municipal d’Investigació Mèdica, and all mothers provided signed informed consent.

### Study variables

The California Preschool Social Competence Scale (CPSCS) (Levine et al. 1969) and the Attention Deficit Hyperactivity Disorder Criteria of Diagnostic and Statistical Manual of Mental Disorders, 4th ed. (ADHD DSM-IV) (American Psychiatric Association 2000) were scored by each child’s teacher. The CPSCS covers a wide range of behaviors such as response to routine, response to the unfamiliar, following instructions, making explanations, sharing, helping others, initiating activities, giving direction to activities, reaction to frustration, and accepting limits. The ADHD DSM-IV checklist comprises 18 items designed to evaluate attention deficit, hyperactivity, and impulsivity in children. To assess 1-month intertest reliability, we randomly selected two small subgroups of children from both cohorts (*n* = 12 and *n* = 34). We used Cohen’s kappa formula to calculate the intertest reliability. We applied the absolute kappa coefficient weighted with the “prerecorded” weights, taking into account the potential missing. An acceptable kappa coefficient of agreement would be > 0.40. The test–retest reliability by kappa formula was satisfactory for the CPSCS (mean = 0.59) and the ADHD checklist (mean = 0.71). The teachers did not know the degree of exposure to OCs. The cognitive skills of the children were also evaluated with the McCarthy Scales for Infant Development as described in previous studies (Ribas-Fitó et al. 2006).

We used a gas chromatograph with electron capture detection (Hewlett Packard 6890N GC-ECD; Hewlett Packard, Avondale, PA) to quantify HCB and other OCs (Carrizo et al. 2006). Quantification was performed using external standards, with the polychlorinated biphenyl (PCB)-142 injection standard used to correct for volume. Recovery of 1,2,4,5-tetrabromobenzene and PCB-209 (75–115%) was used to correct results. Limit of detection was 0.02 ng/mL. A value of 0.01 ng/mL was given for the nonquantifiable concentrations. Serum samples were stored at −40°C until analysis. All the analyses were carried out in the Department of Environmental Chemistry (IIQAB-CSIC) in Barcelona, Spain.

Information on socioeconomic background, maternal diseases and obstetric history, parity, sex, fetal exposure to alcohol (at least two glasses per week during the entire pregnancy) and cigarette smoking (at least one cigarette per day during the last trimester), type and duration of breast-feeding, education, and social class was obtained through questionnaires administered in person after delivery and at 48 months. We used the UK Registrar General’s 1990 classification to group the cohort by social class according to maternal and paternal occupation, coded using the International Standard Classification of Occupations (ISCO-88) (Warwick Institute for Employment Research 2006). Duration of breast-feeding was categorized into four groups: < 2, 2–16, 16–28, and > 28 weeks. Information on maternal diet was obtained through a food-frequency questionnaire administered at the third trimester of gestation.

### Statistical analysis

The CPSCS scores were skewed to the left and were categorized into two subgroups according to a scoring of less or more than 80 points (which represents the social competency female and male mean row score for a 4-year-old child according to the scale). The criterion for ADHD was the presence of either six or more symptoms of inattention or six or more symptoms of hyperactivity–impulsivity. Both components (inattention and hyperactivity) were also studied separately. Cord serum HCB concentrations were categorized into four categories (< 0.5, 0.5–0.99, 1.00–1.49, and > 1.50 ng/mL). We used a natural logarithmic transformed variable when HCB was treated as a continuous variable. We used multivariate models to adjust for the significant covariates reported from the literature, such as maternal and paternal social class and education, mother’s parity and marital status, child’s sex, age, and school season during test administration, cohort, and psychologist. Those variables that altered the HCB coefficient by ≥ 10% in the categorical or continuous models remained in the model. All the models were repeated for each specific cohort. All statistical analyses were conducted with the Stata 8.0 statistical software (StataCorp, College Station, TX, USA).

## Results

HCB was detected and quantified in all cord serum samples from the two cohorts. Concentrations of HCB were, as expected, higher in Ribera d’Ebre, but the maximum values were higher in Menorca (Table 1). Children from the Ribera d’Ebre cohort were more likely to be an only child and to have been breast-fed for shorter periods. The mothers of this cohort were less educated and were more likely to be of lower social class and to drink and smoke during pregnancy than those of the Menorca cohort (*n* = 18). The crude behavioral evaluation was equal in both cohorts (the frequency of a low Social Competence scoring was 19% in Ribera d’Ebre and 23% in Menorca; the frequency of ADHD was 15% in Ribera d’Ebre and 16% in Menorca).

The determinants of *in utero* HCB exposure are shown in Table 2. Children with higher concentrations of HCB in cord blood were more likely to be from the Ribera d’Ebre cohort and to have higher concentrations of PCBs, *p,p*′-dichlorodiphenyldichloroethylene (DDE) and *p,p*′-dichlorodiphenyltrichloroethane (DDT); their parents were older and were of lower socioeconomic class and their mothers were heavier. There were no differences between children with OC measurements (mean Social Competence = 90.7) and those without (mean Social Competence = 90.8) (*p* = 0.98).

Table 3 shows the crude and adjusted associations between HCB and the Social Competence scores. All the categories of HCB exposure were associated with an increase in the risk of having a poorer Social Competence score, but only those children with concentrations of HCB > 1.5 ng/mL at birth had a statistically significant increased risk [relative risk (RR) = 4.04; 95% confidence interval (CI), 1.76–9.58]. The crude and adjusted risks of having an ADHD criteria are shown in Table 4. Children in the highest category of HCB had a 2.71-fold increased risk for ADHD symptoms (95% CI, 1.05–6.96). The two components of ADHD were analyzed separately and the results showed that concentrations of HCB were associated with both inattention [RR for log-transformed HCB in cord serum = 1.82 (95% CI, 1.06–3.14) and for hyperactivity = 1.68 (95% CI, 0.91–3.11)]. The results for the Menorca children only yielded the same results. The results for the Ribera d’Ebre cohort alone were in the same direction but not statistically significant. Concentrations of HCB in the serum of children at 4 years of age were not associated with any of the outcomes.

The Social Competence and the ADHD checklist were highly correlated. Those children without ADHD symptoms had a mean (± SE) score in the California test of 95.21 ± 0.58, whereas those children with ADHD symptoms had a score of 76.18 ± 1.34. The difference was statistically significant with a *p*-value < 0.001.

Prenatal exposure to HCB was not associated with any of the McCarthy scales. Inclusion of the General Cognitive score of the McCarthy Scale in the models did not change the results of the association between HCB and the Social Competence [RR for the highest category of HCB = 4.65 (95% CI, 1.86–11.63)], and HCB and ADHD [RR = 3.20 (95% CI, 1.15–4.12)]. Further adjustment for other OCs such as PCBs, *p,p*′-DDE, and *p,p*′-DDT did not change the results. None of these chemicals were associated with Social Competence or ADHD.

## Discussion

Prenatal exposure to HCB is associated with a decrease in the Social Competence test performance and an increase of ADHD symptoms at 4 years of age. This association is statistically significant only at higher categories of exposure. Children with HCB concentrations in cord serum > 1.5 ng/mL had a 4-fold increase of having a poor Social Competence score and a 2.7-fold increase of having ADHD symptoms. No association was found between HCB and the cognitive and psychomotor performance of these children.

The evidence of neurotoxicity in humans exposed to HCB was provided by studies of people in southeast Turkey who consumed contaminated bread in the late 1950s. Neurologic symptoms included loss of appetite, tremors, convulsions, and weakness (Peters et al. 1982). Follow-up studies found that neurologic symptoms persisted in adults who had been exposed as children. During the grain poisoning epidemic, there was an extremely high (95%) rate of mortality in infants < 2 years of age who had been breast-fed by mothers who had ingested the contaminated bread; these children exhibited convulsions, tremors, and progressive weakness before death (Peters et al. 1966). A recent study described a significant association between cord blood HCB and one of the blocks of the Neurobehavioral Evaluation System Continuous Performance Test, which evaluates sustained attention, at 8 years of age (Stewart et al. 2005), but further adjustment for covariates has found the association nonsignificant. The authors also found an adjusted association between prenatal PCB exposure and an impaired response inhibition at 9.5 years of age, confirming what they had described at 4.5 years of age (Stewart et al. 2003). We did not find any association between PCBs or *p,p*′-DDE and the assessed behavioral patterns.

Information about the neuronal effects of HCB is limited, despite the fact that after acute exposure animals die because of neurotoxic symptoms [International Agency for Research on Cancer (IARC) 1979]. Reported neurotoxic effects in rats exposed to HCB are hyperexcitability, tremors, weak legs, and paresis (De Matteis et al. 1961; Vos et al. 1971), an increase in the activity level (Goldey and Taylor 1992), and changes in operant behavior in adult rats (Lilienthal et al. 1996), but no significant effects on learning or motor activity have been detected.

The mechanisms by which HCB may cause behavioral impairments are not known. It has been suggested that HCB interferes with myelination during development (Goldey and Taylor 1992). Alterations in regional brain concentrations of serotonin, dopamine, and norepinephrine have also been described by Bleavins et al. (1984), and a recent study has found that exposure to HCB can produce oxidative stress and that the brain is a sensitive target organ of HCB toxicity (Song et al. 2006). No signs of histopathologic changes in the brain, spinal cord, motor and sensory nerves, and skeletal muscles have been found (Campbell 1963; Kuiper-Goodman et al. 1977).

It is difficult to elucidate why the HCB effects were apparent only on the behavioral and not the cognitive functions. A study investigating the potential effects of consuming fish from the Great Lakes was unable to correlate HCB levels in umbilical blood or breast milk with infant intelligence test results (Darvill et al. 2000). In a previous study with children from the Ribera d’Ebre cohort, we reported no association between prenatal exposure to HCB and the mental and psychomotor functions of the children at 13 months of age (Ribas-Fitó et al. 2003), although no behavioral test batteries were performed. Most research on OCs and neurodevelopment has relied on broad measures of global cognitive functioning. These tests have not been designed to measure specific neurobehavioral processes, such as attention and social behavior. In the present study, we also did not find any association between HCB and the cognitive skills in the two cohorts at 4 years of age (Ribas-Fitó et al. 2006). The association was significant only when the behavioral domains such as the Social Competence and ADHD were studied. ADHD is a neuropsychiatric disorder characterized by pervasive inattention and/or hyperactivity–impulsivity and resulting in significant functional impairment. The Centers for Disease Control and Prevention estimate that 4.4 million youth 4–17 years of age have been diagnosed with ADHD by a health care professional, and as of 2003, 2.5 million youth 4–17 years of age are currently receiving medication treatment for the disorder (Lesesne et al. 2000).

A potential limitation of the present study is the nonresponse rate (20.6%). Subjects were not included (*n* = 98) because no information could be obtained from the schools. The nonresponse rate is unlikely to be related to HCB levels or the Social Competence scores of the children. One of the cohorts in the present study was studied in an area where HCB is the main pollutant, deriving from an electrochemical factory in the vicinity, and the population living in this area has the highest levels of HCB ever reported for nonoccupational exposure (Sala et al. 2001). The Menorca cohort represents a general population with little industrial activity. Geographic differences between Menorca and Ribera d’Ebre were eliminated by analyzing the data by cohort. The results using the Menorca cohort alone showed no differences compared with the two-cohort analyses, and the results from the Ribera d’Ebre cohort alone were in the same direction but not statistically significant. This lack of significance could be explained by the small size of the cohort. Because Menorca has no local pollution sources, the results encountered here could be representative of other areas where several years have elapsed since HCB use was banned. Residual confounding was minimized by the adjustment for the potential confounders.

Overall, prenatal exposure to current concentrations of HCB is associated with a decrease in the Social Competence scores and an increase of the ADHD symptomatology at preschool ages. These results suggest that some infants may be at risk for developing neurotoxicity from HCB due to relatively high concentrations of HCB detected in cord serum and breast milk from women in certain parts of the world.

## Figures and Tables

**Table 1:** Distribution (median and 25th and 75th percentiles) of HCB in cord serum (ng/mL) by cohort.

Cohort	No.	Minimum	25th	Median	75th	Maximum
Total	475	0.14	0.48	0.73	1.13	9.82
Ribera d’Ebre	70	0.17	0.80	1.13	1.69	5.77
Menorca	405	0.14	0.46	0.68	1.02	9.82

**Table 2:** Distribution of child, paternal, and maternal variables according to concentrations of HCB in cord serum (*n* = 405).

	HCB category (ng/mL)				
Variable	< 0.5	0.5–0.99	1–1.49	≥ 1.5	*p*-Trend
Child variables					
Female sex (%)	53	51	50	41	NS
Population (%)					
Menorca					
Ciutadella	47	43	32	28	
Maó	16	19	22	15	
Other	31	27	21	24	
Ribera d’Ebre					
Flix	2	4	15	23	
Other	4	7	10	10	< 0.001
Gestational age (weeks)	39.4	39.5	39.4	39.7	NS
Birth weight (g)	3164.4	3259.3	3125.0	3290.5	NS
Breast-feeding, yes (%)	84	83	79	78	NS
Age at examination (years)	4.59	4.59	4.58	4.57	NS
No. of siblings (%)					
None	34	29	29	22	
1	50	52	53	63	
> 1	16	19	18	15	NS
PCBs (ng/mL)	0.64	0.86	0.90	0.90	0.032
*p,p*′-DDE (ng/mL)	0.10	0.18	0.17	0.28	< 0.001
*p,p*′-DDT (ng/mL)	0.93	1.58	1.97	2.44	< 0.001
Paternal variables					
Age (years)	30.5	32.0	33.4	34.1	< 0.001
Years of education (%)					
> 16	5	7	16	10	
12–15	22	33	25	28	
8–11	59	51	50	49	
< 8	14	9	9	13	NS
Social class (%)					
Professional	10	17	24	18	
Skilled	73	67	57	48	
Partially skilled	17	16	19	34	0.003
Maternal variables					
Age (years	27.2	29.1	30.4	30.8	< 0.001
Height (cm)	161.2	160.9	161.1	160.3	NS
Weight (kg)	56.3	59.5	60.6	63.4	< 0.001
Years of education (%)					
> 16	10	16	10	7	
12–15	24	29	40	29	
8–11	53	47	34	46	
< 8	13	8	16	18	0.048
Social class (%)					
Professional	10	13	23	10	
Skilled	44	57	34	34	
Partially skilled	22	12	17	24	
Unemployed	24	18	26	32	0.001
Alcohol, yes (%)	22	28	23	21	NS
Tobacco, yes (%)	33	15	28	28	0.002
Food consumption during pregnancy (servings per week)					
Vegetables	11.1	11.0	12.1	11.9	NS
Pasta and/or rice	3.5	3.4	3.1	3.3	NS
Cooked meat	5.1	4.8	5.1	5.7	NS
Processed cold meat	3.7	3.8	4.0	4.3	0.09
Fish	1.6	1.6	1.7	1.4	NS
Seafood	1.1	1.3	1.1	1.0	NS
Dairy food	27.5	26.0	26.6	25.1	NS
Eggs	2.6	2.5	2.6	2.3	NS

NS, not statistically significant.

**Table 3:** Crude and adjusted RR of scoring < 80 points in the Social Competence scale at 4 years of age in relation to *in utero* exposure to HCB [coefficient (95% CI)].

Exposure	Unadjusted (*n* = 377)	Adjusted^a^ (*n* = 377)	Adjusted for other OCs^b^ (*n* = 377)	Menorca cohort^b^ (*n* = 329)
HCB category				
Reference^c^	1	1	1	1
0.5–0.99 ng/mL	1.16 (0.62–2.18)	1.40 (0.68–2.87)	1.77 (0.83–3.79)	1.84 (0.82–4.11)
1–1.49 ng/mL	1.04 (0.48–2.29)	1.47 (0.59–3.62)	1.83 (0.72–4.69)	1.51 (0.52–4.35)
≥ 1.5 ng/mL	2.88 (1.39–5.97)*	4.04 (1.76–9.58)*	5.63 (2.13–14.88)*	6.18 (2.06–18.50)*
HCB (ng/mL)^d^	1.52 (1.05–2.22)*	1.79 (1.15–2.76)*	2.10 (1.30–3.40)*	2.18 (1.28–3.74)*

a Adjusted for age, cohort, sex, maternal education, paternal education, tobacco and alcohol exposure, maternal age in years, and type and duration of breast-feeding (see “Population and Methods”).

b Adjusted for same variables above and PCBs, *p,p*′-DDE and *p,p*′-DDT.

c Reference group: < 0.5 ng/mL.

d Natural log-transformed HCB concentration.

* *p* < 0.05.

**Table 4:** Crude and adjusted RR of having ADHD symptoms at 4 years of age in relation to *in utero* exposure to HCB [coefficient (95% CI)].

Exposure	Unadjusted (*n* = 377)	Adjusted^a^ (*n* = 377)	Adjusted for other OCs^b^ (*n* = 377)	Menorca cohort^b^ (*n* = 329)
HCB category				
Reference^c^	1	1	1	1
0.5–0.99 ng/mL	1.19 (0.58–2.42)	1.23 (0.54–2.78)	1.47 (0.63–3.46)	1.38 (0.57–3.32)
1–1.49 ng/mL	1.73 (0.77–3.91)	2.28 (0.88–5.96)	2.74 (1.01–7.45)*	2.17 (0.73–6.49)
≥ 1.5 ng/mL	2.05 (0.90–4.67)**	2.71 (1.05–6.96)*	3.43 (1.24–9.51)*	3.11 (1.01–9.55)*
HCB (ng/mL)^d^	1.49 (0.99–2.24)**	1.63 (1.02–2.63)*	1.88 (1.13–3.14)*	1.77 (1.00–3.11)*

a Adjusted for age, cohort, sex, maternal education, paternal education, tobacco and alcohol exposure, maternal age in years, and type and duration of breast-feeding (see “Population and Methods”).

b Adjusted for same variables above and PCBs, *p,p*′-DDE and *p,p*′-DDT.

c Reference group: < 0.5 ng/mL.

d Natural logarithmic transformed HCB concentration.

* *p* < 0.05

** *p* < 0.10.
